# Injury alters motivational trade-offs in calves during the healing period

**DOI:** 10.1038/s41598-021-86313-z

**Published:** 2021-03-25

**Authors:** Sarah J. J. Adcock, Cassandra B. Tucker

**Affiliations:** 1 Center for Animal Welfare, Department of Animal Science, University of California, Davis, 95616 USA; 2 Animal Behavior Graduate Group, University of California, Davis, 95616 USA

**Keywords:** Behavioural ecology, Behavioural methods

## Abstract

Injury can produce long-lasting motivational changes that may alter decisions made under risk. Our objective was to determine whether a routine painful husbandry procedure, hot-iron disbudding, affects how calves trade off risk avoidance against a competing motivation (i.e., feeding), and whether this response depends on time since injury. We used a startle test to evaluate this trade-off in calves disbudded 0 or 21 days previously and non-injured control calves. For 3 days, calves were individually habituated to the testing arena in which they received a 0.5 L milk meal via a rubber teat. On the 4^th^ day, upon approaching the milk reward, the calf was startled by a sudden noise. We assessed the duration and magnitude of the calf’s startle response, their latency to return to the milk bottle, and duration spent suckling after startling. No treatment differences were observed in the duration and magnitude of the startle response or in the probability of returning to the bottle after startling. However, among those who did return, disbudded calves spent longer suckling, indicating they accepted more risk in order to feed compared to controls. In addition, calves with 21-day-old injuries tended to return to the bottle faster compared to newly disbudded calves and controls. We suggest that hot-iron disbudding increases calves’ motivation to suckle, as they were more likely to prioritize this behaviour over risk avoidance compared to control calves. This effect was most evident 21 days after disbudding, indicating that injury can produce long-term changes in motivational state.

## Introduction

Many behavioural decisions produce a trade-off where an animal can gain rewards by engaging in one behaviour (e.g., feeding), but at the cost of exposure to increased risk (e.g., predation). In general, when perceived risk is high, the defensive motivational circuit in the brain should inhibit other motivational systems that may compromise safety (e.g., hunger), and vice versa when risk is low^1^. When activated, the defensive motivational circuit organizes a cascade of behaviours that are optimized to mitigate risk, prompting startle or orientation responses towards a potential threat and culminating in flight or fight actions if attack is imminent^2^.

Following sub-lethal injury, an animal may incur increased predation risk, as predators preferentially prey on compromised individuals^3,4^. Selection pressures should therefore favour behavioural decisions that offset this risk, increasing chances of a successful recovery. Accordingly, more defensive behaviour has been observed in injured invertebrates including squid^5,6^, crayfish^7^, moth larvae^8^, and flies^9^ compared to uninjured controls, and may persist for weeks^9^. This long-lasting behavioural sensitization improves survival of injured animals during predatory encounters^4^. To our knowledge, the effect of injury on defensive behaviour in vertebrates has not been explored.

Farm animals routinely undergo injurious husbandry procedures, but the long-term impact on the animal’s motivational state and subsequent decisions is poorly understood. In the dairy industry, a common husbandry practice is to prevent horn growth by cauterizing the horn-growing tissue with a heated iron when calves are 0–2 months of age. This procedure, known as hot-iron disbudding, is performed on 94% and 81% of dairies in the U.S. and E.U., respectively, to reduce injuries to humans and other animals^10,11^. Disbudding causes acute behavioural and physiological pain responses^12^. The resulting burns take 6 to 13 weeks to re-epithelialize^13,14^, and remain sensitive to mechanical stimulation throughout this time, if not longer^13,15^. Calves display a negative judgment bias consistent with risk aversion 22 h after disbudding^16^, but it is unknown how long this bias persists. We recently demonstrated that calves given analgesia 11 days after disbudding show behavioral changes consistent with reduced pain^17^, and 3 weeks after disbudding, calves are more likely to choose an environment paired with analgesia than their non-disbudded counterparts^18^. Together, these findings indicate that calves continue to experience pain during the healing period even when the wounds are not being stimulated. It is possible that, in addition to ongoing pain, the weeks following disbudding are characterized by motivational changes consistent with risk-averse decision-making.

Our objective was to determine whether disbudding affects how calves trade off risk avoidance against a competing motivation (i.e., feeding), and whether this response depends on the time since injury. We used an acoustic startle paradigm to determine whether disbudded calves exhibited more defensive behaviour (i.e., startle response) and an accompanying reduction in risky behaviour (i.e., feeding) in the immediate hours after disbudding, and 3 weeks later during healing. We have previously used this paradigm to demonstrate that calves were more risk-averse in the presence of a predator odour, startling more to an acoustic stimulus and delaying return to a milk reward, compared to calves exposed to non-threatening odours^19^. Thus, we predicted that compared to non-injured controls, disbudded calves would (1) have a higher magnitude and duration of the startle response, (2) take longer to return to a milk reward and spend less time suckling after startling, and that (3) this effect would be greatest in the immediate hours after disbudding, but continue to persist during the healing period.

## Materials and methods

This work was undertaken at the University of California Davis Dairy Teaching and Research Facility from June to September 2018. All experimental protocols were approved by and carried out in accordance with the University of California Davis Institutional Animal Care and Use Committee (protocol # 20505).

### Treatments

We enrolled all female calves born between June 19 and September 1 2018, for a total of 28 Holsteins and 8 Jerseys. Our sample size was determined by the availability of calves being born in our herd of approximately 105 lactating cows during this period. Calves were blocked by birth order and randomly allocated to 1 of 3 treatments balanced for breed: disbudded the morning of (Day 0) or 21 days before (Day 21) the startle test, or sham-disbudded (Sham, n = 12/treatment). Among the control calves, half were sham-disbudded the morning of the test, whereas the other half underwent the procedure 21 days earlier. Birth weights were similar across treatments (mean ± SD; Day 0: 35 ± 5 kg; Day 21: 35 ± 6 kg; Sham: 36 ± 9 kg). The startle test occurred between 25 and 32 days of age for all calves. Thus, all Day 0 calves and half of the Sham calves were disbudded between 25 and 32 days of age, and all Day 21 calves and half of the Sham calves were disbudded between 4 and 11 days of age. This design meant all animals were at the same stage of cognitive and motor development during data collection. This was a priority for us because we expected age to strongly influence behavioural responses during the startle test. While it is also possible that disbudding at different ages may affect responses, previous research suggests disbudding has similar outcomes across this range^13,15,20^.

### Animal husbandry and housing

Immediately after birth, calves were housed individually in outdoor enclosures consisting of a plastic hutch (2.0 m long × 1.5 m wide) and a wire-fenced pen (2.0 m long × 1.5 wide × 0.9 m high). The enclosures were spaced 0.5 m apart and bedded with sand approximately 15 to 20 cm deep.

Calves were bottle-fed colostrum twice a day for 5 days. From 5 days of age, calves received milk replacer (26% CP and 16% fat, 15% total solids; Calva Products Inc., Acampo, CA) in bottles at 0645, 1245, and 1845 h. At each meal, Holsteins were fed 1.9 L from 1 to 13 days, 2.4 L from 14 to 23 days, and 2.8 L from 24 days. Jerseys received 1.4 L from 1 to 13 days, 1.9 L from 14 to 23 days, and 2.4 L from 24 days. Water and starter (18.3% CP, 2.8% fat, 4% crude fat; Associated Feed & Supply Co., Turlock, CA) were provided ad libitum in buckets. As part of a separate concurrent study, 11 calves (3 Sham, 3 Day 21, 5 Day 0) received chopped mountain grass hay (34% CP) ad libitum.

### Disbudding

Disbudding occurred between 730 and 1000 h. For the procedure, the calf was restrained in a head device in her home enclosure^21^. A 5 × 5 cm patch of hair was clipped with a size 40 electric razor blade on each side of the head to locate the horn bud. We used a 20 gauge × 25 mm needle to administer a cornual nerve block consisting of 5.5 mL buffered lidocaine (2% lidocaine hydrochloride diluted with 8.4% sodium bicarbonate in a 10:1 ratio). If the horn bud was not numb after 10 min, as assessed by pinprick, we gave an additional 2 mL of buffered lidocaine (13% of horn buds). An electric cautery iron (X50, Rhinehart Development Corp., Spencerville, IN) was fitted with a 1.3 cm tip and heated to 439 ± 15 °C (mean ± SD). It was applied to the horn bud for 17 ± 5 s (mean ± SD). Immediately before disbudding, the calf received approximately 1 mg/kg of meloxicam tablets in a gelatin capsule (3.5 g; Torpac Inc., Fairfield, NJ). For Day 0 calves, meloxicam was given after the startle test had occurred later that same day (maximum 12 h later) to ensure the calf was in a drug-free state during the test. Sham-disbudded calves received the same treatment, with the exception that the iron was ambient temperature and the gelatin capsule was empty. Sham calves did not receive meloxicam because the Animal Medicinal Drug Use Clarification Act limits nontherapeutic off-label use of this drug^22^. SJJA performed all disbudding procedures.

### Arena

**Figure 1:**
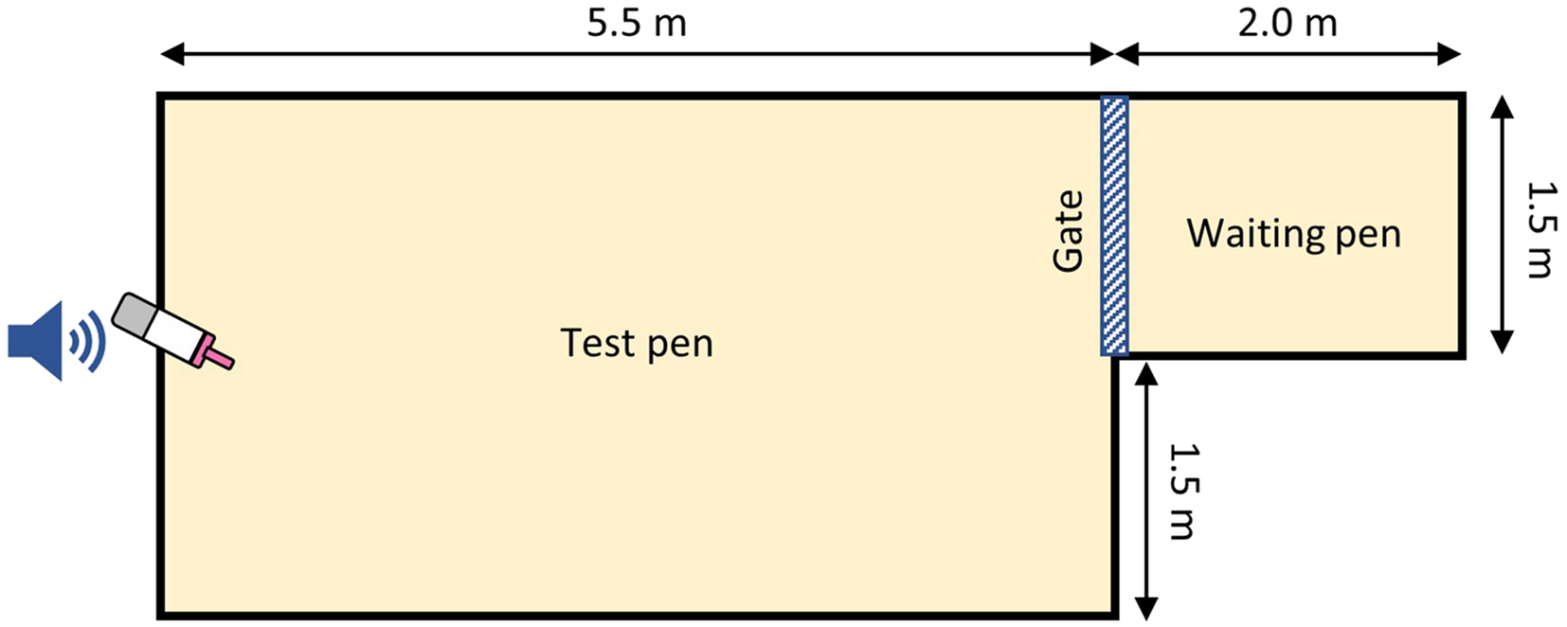
Aerial view of the arena used for startle tests, including the position of the milk bottle and speaker used to broadcast the startle noise. Figure is drawn to scale.

We tested calves individually in a single 10-min period in a shaded outdoor arena bedded with 10 to 15 cm of sand. The arena was divided into a waiting pen (2.0 × 1.5 m) and a test pen (3.0 × 5.5 m) constructed of 0.9 m high wire panels (MidWest Homes for Pets Foldable Metal Exercise pen, Muncie, IN). A rolling gate provided access between the pens (Fig. 1).

A bottle containing 500 mL of the calves’ regular milk replacer was secured to the panel opposite the entrance to the test pen. The bottle was fitted with a rubber teat positioned 80 cm above the ground. Between calves, a fresh bottle was placed in the arena and urine and feces were removed with a shovel.

### Testing procedure

Calves were habituated to the arena for 15 min daily between 700 and 1100 h for 3 consecutive days before the startle test. Calves were brought to the arena in the same order each day, with order balanced across treatments. During habituation, no startle stimulus was delivered, but otherwise the same procedure followed on test days was applied.

The startle test occurred between 1530 and 1800 h (Supplementary Video S1). The calf was transported from her home pen to the waiting pen in a cart (Caf-Cart, Raytec, Ephrata, PA). The test began when the gate providing access to the test pen was opened and ended after 10 min. The gate was closed behind the calf after she had entered so that the waiting pen was inaccessible during the test. Three observers were seated quietly 3.5 m away from the pen during the test, and were partially concealed behind a tree branch. One observer remotely controlled the speaker broadcasting the startle noise, while the other two observers were present to respond if a calf escaped from the arena (only one calf jumped out, on the first day of habituation, and was promptly escorted back into the pen). Calves showed no apparent responses to the observers and had no visual contact with other animals.

As soon as the calf’s mouth was within a tongue’s reach of the teat, a 0.4 s, 105 ± 2 dB burst of white-noise was emitted through a wireless speaker (OT4200 Big Turtle Shell, Outdoor Tech, Laguna Hills, CA) mounted directly behind the bottle. The noise was created using an online signal generator^23^. We measured the sound level using a decibel meter (BAFX Products, Milwaukee, WI) held 30 cm in front of the bottle, approximating the distance of the calf’s ears to the source.

### Behavioural data collection

Tests were recorded with a camcorder (HC-V180, Panasonic, Kadoma, Japan) positioned on a tripod approximately 3 m away from of the pen. One trained observer, blinded to the treatments, scored behaviours in all videos taken of the startle test and the third day of habituation (Table 1). Videos were analysed using BORIS (Behavioural Observation Research Interactive Software^24^). Intra-observer reliability was calculated using 12 randomly selected videos of the startle test (Intraclass correlation coeffcient ≥ 0.95).

**Table 1:** Behavioural definitions used to evaluate calves’ responses in an arena test.

Tissue	Definition
Latency to approach	Time until calf is within a tongue’s reach of the teat
Startle duration	The time between the noise playing and when the calf has all 4 feet on the ground or makes at least a quarter of a turn back towards the bottle
Latency to return	Time from startle stimulus delivery until calf begins suckling
Duration spent suckling	The mouth is latched onto the teat and the jaw is moving. A suckling bout ends when the mouth is off the teat for at least 5 s

As soon as the calf approached a milk bottle fitted with a rubber teat, a sudden noise was emitted to induce a startle response. The day before the startle test, the calf underwent a third and final period of habituation, in which the milk bottle was present, but no noise was emitted. Therefore, only latency to approach and duration spent suckling were scored during the habituation period.

Accelerometers (Hobo Pendant G Acceleration Data Logger, Onset Computer Corporation, Bourne, MA) were used to assess the magnitude of the startle response. On habituation and test days, we fitted calves with a triaxial accelerometer set to record acceleration in the x-, y-, and z-axis every 0.05 s. The accelerometer was placed in a pouch, strapped around the right hind leg, and secured with Vet Wrap (Co-Flex, Andover Coated Products Inc., Salisbury, MA) while the calf was in the waiting pen of the arena, immediately before the gate to the test pen was opened. Data were downloaded using HOBOware Pro Software (Onset Computer Corporation, Bourne, MA). To calculate the magnitude of the startle response, we summed total acceleration in all 3 axes over the startle duration for that calf. Total acceleration was calculated as the square root of the sum of squared acceleration in each axis^25^. No startle response was recorded for one calf who did not approach the bottle on the test day.

All calves were weighed the morning of the startle test (mean ± SD; Day 0: 56 ± 10 kg; Day 21: 55 ± 9 kg; Sham: 55 ± 11 kg).

### Wound healing and sensitivity

We measured sensitivity via mechanical nociceptive thresholds around the horn bud area 1 to 2 h after the startle test using a digital algometer fitted with a 4-mm-diameter round rubber tip (ProdPlus; TopCat Metrology Ltd., Little Downham, UK). The calf was restrained in the head device in her home pen and blindfolded to reduce responses to visual cues. We then applied an increasing amount of force to the edge of the disbudding wound, or intact horn bud for sham calves, as described previously^13^. The test ended when the calf moved her head or a maximum cut-off point of 10 N was reached. We repeated the test if a fly landed on the head, a loud noise occurred, or the calf urinated or defecated. If a test was interrupted 3 times, it was abandoned (0% of tests).

Wound sensitivity was tested at the lateral and caudal edges of each wound or the equivalent location on sham calves. The order of test sites was: left lateral, left caudal, right caudal, and right lateral. To ensure force was applied at a consistent rate, personnel operating the algometer were trained and met a set of rigorous criteria before performing the tests^13^. We calculated the rate that force was applied in each test from video recordings (0.29 ± 0.10 N/s; 2% of videos missing). If force was increased at a rate < 0.1 or > 0.6 N/s or video was missing, the data were excluded (3% of tests). Due to the nature of the tests, the operator of the algometer was not blind to treatment.

We took digital photographs of the wound with a DSLR camera (D5300; Nikon Corp., Tokyo, Japan) after sensitivity testing was completed. Photos were taken 15 cm from the wound. One person scored the photos for tissues present in the wound bed using a 0/1 scoring system^13^. Due to the clear differences in Day 0 and Day 21 wounds, the scorer was not blind to treatment.

### Statistical analysis

Due to the presence of zeros in the data, we used zero-inflated beta regressions to assess the effect of treatment (Sham, Day 0, Day 21) on the proportion of time suckling on the third day of habituation and during the startle test. A zero-inflated beta regression is a mixture of two models: a beta model for estimating non-zero proportions and a logistic model for estimating the probability of zeroes^26^. This approach allowed us to infer treatment effects on both the occurrence and duration of suckling. General linear models were used to test the effect of treatment on the duration of the startle response and its magnitude as measured from the accelerometer data.

We analyzed the effects of treatment on latency to approach the bottle and latency to return after startling using parametric survival regression models with a log-logistic distribution. Days on which the calf did not perform the behaviour within the allotted time (15 min for habituation, 10 min for startle test) were handled as right-censored data.

We ran a general linear model to test the effect of treatment on wound sensitivity. A preliminary analysis indicated that there was no effect of side (left vs right) or location (caudal vs lateral) on wound sensitivity, so we averaged data for each calf into one score.

Data were analysed in R, version 3.5.2^27^. General linear models were fitted using the “lm” function in base R. We confirmed homogeneity of variance and normality using residuals vs fits plots and Q-Q plots, respectively. Beta and survival regressions were performed with the “glmmTMB” function in the *glmmTMB* package version 1.0.0^28^, and the “survreg” function in the *survival* package version 2.38^29^, respectively. If treatment effects were identified in any of the models (*P* < 0.1), we calculated pairwise contrasts using Tukey’s method with the *emmeans* package, version 1.4.5^30^.

## Results

### Behaviour

#### Habituation

We observed no differences in latency to approach the bottle or duration spent suckling between treatments on the third, and final, day of habituation (Table 2). The logistic component of the zero-inflated beta regression indicated no treatment differences in the probability of suckling on the final day of habituation (*X*^2^_2_ = 0, *P* = 1).

**Table 2:** Estimated marginal mean ± SE of behaviours in calves disbudded the morning of (Day 0) or 21 days before (Day 21) the startle test, or sham-disbudded (Sham, n = 12/treatment).

Measure	Treatment			Test statistic	P-value
	Sham	Day 0	Day 21		
**Day 3 Habituation**					
Latency to approach (s)	28 ± 11	17 ± 6	23 ± 9	*X*^2^_2_ = 0.89	0.641
Proportion of time suckling^1^	0.18 ± 0.02	0.17 ± 0.02	0.24 ± 0.03	*X*^2^_2_ = 4.51	0.105
**Startle test**					
Latency to approach (s)	12 ± 3	18 ± 6	11 ± 3	*X*^2^_2_ = 1.49	0.476
Startle duration (s)	2.4 ± 0.2	2.6 ± 0.2	2.8 ± 0.2	*F*_2, 32_ = 0.80	0.460
Startle magnitude (g)	53 ± 5	57 ± 5	62 ± 5	*F*_2, 32_ = 1.04	0.364
Latency to return (s)	63 ± 37^a^	85 ± 56^a^	12 ± 5^b^	*X*^2^_2_ = 7.47	0.024
Proportion of time suckling^1^	0.13 ± 0.02^a^	0.24 ± 0.02^b^	0.22 ± 0.02^b^	*X*^2^_2_ = 15.13	< 0.001

The startle test consisted of a 10-min period in which a sudden noise was emitted as soon as the calf approached the milk bottle. The day before the startle test, the calf underwent a third and final 15-min period of habituation, in which the milk bottle was present, but no noise was emitted (Day 3 Habituation). Different superscripts indicate treatments differ (*P* ≤ 0.080).

^1^Estimates are based on the beta regression component of the zero-inflated beta regression model. Thus, zero values are excluded.

#### Startle test

**Figure 2:**
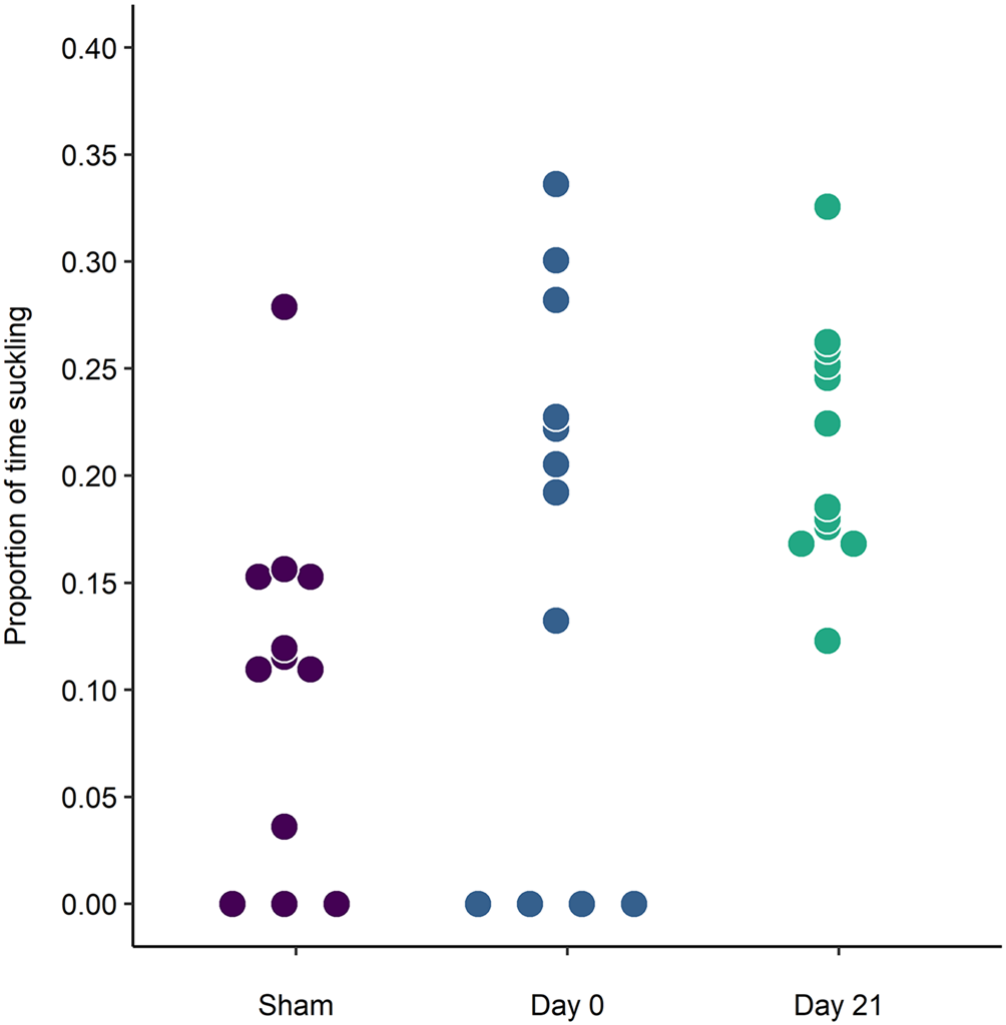
Scatterplot showing the proportion of time spent suckling from a milk bottle in calves disbudded the morning of (Day 0) or 21 d before (Day 21) the startle test, or sham-disbudded (Sham, n = 12/treatment). The startle test consisted of a 10-min period in which a sudden noise was emitted as soon as the calf approached the milk bottle. Each dot represents an individual calf.

On the day of the startle test, calves were quick to approach the bottle across treatments (Table 2). One Day 0 calf did not approach the bottle, and so did not receive a startle test. No treatment differences in the duration of the startle response or its magnitude, as measured from the accelerometer data, were observed (Table 2). Following startle, 3 Sham and 3 Day 0 calves did not return to the bottle. All Day 21 calves returned (Fig. 2). However, these treatment differences in the probability of suckling after startle were not statistically significant (*X*^2^_2_ = 0.201, *P* = 0.905). Among those who returned to the bottle, Day 0 and Day 21 calves suckled nearly twice as long as the Sham calves. Day 21 calves tended to return 5 to 7 × faster after startling compared to the other 2 groups (Table 2).

### Wound healing and sensitivity

The necrotic tissue was still attached to the scalp in all wounds after the startle test in Day 0 calves. In Day 21 calves, the necrotic tissue was detaching from the scalp in 54% of the wounds and had fallen off in the other 46% (Table 3).

**Table 3:** The percentage of disbudding wounds in which the tissue was present in calves (n = 12, each with 2 horn buds) disbudded 0 (n = 24 wounds) or 21 days (n = 24 wounds) previously.

Tissue	Day 0	Day 21
Attached necrotic	100	0
Burns outside the ring	29	0
Detaching necrotic	0	54
Exudate	0	8
Granulation	0	25
Crust	0	42
Epithelium	0	0

We observed a treatment effect on wound sensitivity (Day 0: 1.01 ± 0.18 N; Day 21: 1.28 ± 0.18 N; Sham: 1.96 ± 0.18 N; *F*_2,33_ ≥ 7.00; *P* = 0.003). Day 0 and Day 21 disbudded calves were more sensitive to pressure algometry compared to Sham calves (*P* = 0.003 and *P* = 0.035, respectively), but did not differ from each other (*P* = 0.575).

## Discussion

We assessed whether hot-iron disbudding, a painful routine husbandry procedure in the dairy industry, affects how calves trade off risk avoidance against a competing motivation (i.e., feeding). In the absence of a threat, all calves were highly motivated to feed, approaching the bottle within seconds. After delivery of the startle stimulus, there were no treatment differences in the probability of returning to the bottle, but Day 21 calves tended to return 5 to 7 × faster than Sham and Day 0 calves. The faster return in Day 21 calves suggests they were more willing to accept risk in order to suckle compared to non-injured controls and recently disbudded calves.

Among the calves who returned to the bottle, both Day 0 and Day 21 calves suckled longer than the Sham calves. This contradicted our prediction that injury would increase risk avoidance at the expense of decreased feeding. It is possible that pain, which persists for several weeks after disbudding^13,15,17,18^, was a motivating factor driving this decision. Indeed, disbudded calves were almost twice as sensitive around their wounds compared to Sham calves 3 weeks after the procedure, which is a replicated and consistent finding in the literature^13,15^. Thus, the greater amount of suckling may have been due to the analgesic and soothing effects elicited by the gustatory and tactile stimulation^31^. Nutritive and non-nutritive suckling ameliorates pain and stress in rat pups and human infants^31–33^, and induces restfulness in calves^34^ and piglets^35^. Compared to healthy controls, rat pups with inflammatory pain nurse more from their dams^36^. The Day 21 calves had longer to form an association between suckling and pain mitigation, which may explain why they returned to the bottle faster than Day 0 calves, who had only one milk meal between disbudding and the startle test. It is also possible that Day 21 is characterized by more pain relative to Day 0; at this time point the initial necrotic tissue was either detaching or had completely fallen off in all wounds, exposing the underlying granulation tissue, which agrees with previously reported healing timelines for disbudding^13,18^. However, although the Day 0 calves were slower to return to the bottle than Day 21 calves, those that did return suckled longer than the Sham calves, suggesting this behaviour could have immediate reinforcing benefits after a painful procedure.

Alternatively, it is possible the disbudded calves suckled more upon returning to the bottle in order to meet the increased energy requirements for wound healing^37^. We think this possibility is less likely, as all control and disbudded calves that returned to the bottle finished their meal, usually within the first minute, and any suckling beyond this time was non-nutritive (pers. obs.). Pharmacological studies with analgesics or appetite suppressors would provide more conclusive evidence as to whether the increased motivation to suckle is a response to pain or hunger due to increased energetic demands.

**Figure 3:**
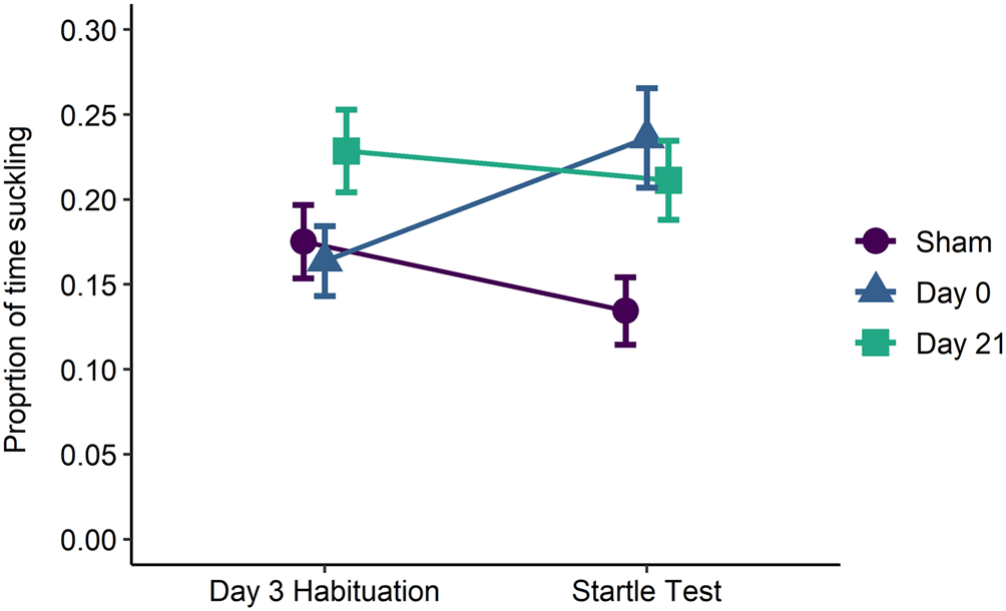
Proportion of time (estimated marginal mean ± SE) spent suckling from a milk bottle in calves disbudded the morning of (Day 0) or 21 d before (Day 21) a 10-min startle test, or sham-disbudded (Sham, n = 12/treatment). The day before the startle test, the calf underwent a third and final 15-min period of habituation, in which the milk bottle was present, but no noise was emitted (Day 3 Habituation). We performed a post-hoc analysis to determine whether the proportion of time suckling differed between the third day of habituation and the startle test for each treatment using a zero-inflated mixed beta regression. The interaction between treatment (Sham, Day 0, Day 21) and period (Day 3 Habituation and Startle test) was included as the fixed effect and calf as the random effect. From the third day of habituation to the startle test, the proportion of time spent suckling tended to decrease in Sham calves (*P* = 0.058), increased in Day 0 calves (*P* = 0.005), and did not change in Day 21 calves (*P* = 0.431). Estimates are based on the beta regression component of the zero-inflated beta regression model. Thus, zero values are excluded.

Calves did not differ in their suckling behaviour on the third day of habituation, when no threat was present. During habituation, the cost to access the teat was low relative to the startle test, and consumption of this resource was similar across treatments. When the cost to access this resource increased with the addition of the startle stimulus, among calves who returned to the bottle, Sham calves tended to suckle less, Day 21 calves maintained their level of use, and Day 0 calves suckled more compared to the final day of habituation (posthoc analysis; Fig. 3). These changes suggest that disbudded calves place a higher value on suckling, potentially due to its soothing or analgesic properties, as they accept more risk to receive the milk reward compared to Sham calves. This increased motivation to suckle is only revealed when calves are forced to prioritize behaviours (i.e., risk avoidance vs feeding). This result, in agreement with theory^38^, suggests that staging trade-offs can identify an animal’s motivational state that is otherwise not apparent from observing uninterrupted behaviour.

In contrast to our prediction, the calf’s startle response to the acoustic stimulus was similar across treatments. The acoustic startle stimulus prompted a flight response lasting 1 to 4 s in all calves, indicating that the noise was processed as a threat. Using the same paradigm as in the present study, we have previously shown that a threatening stimulus, predator odour, increases the acoustic startle response in calves compared to odour of water or an unfamiliar herbivore^19^. The startle magnitudes observed in the previous study were 3 × higher than those reported here due to methodological differences in how startle was defined. We predicted that injury would have a similar effect on the startle response as predator odour, given that defensive behaviours are increased in injured invertebrates^5–9^ and that humans experiencing chronic pain have an increased acoustic startle response^39,40^. However, we did not observe an effect of disbudding on the startle magnitude and duration. To our knowledge, this is the first study to use the startle response to evaluate risk aversion in a non-human animal experiencing pain.

The startle response is modulated by the animal’s preceding motivational state, with negative states potentiating it and positive states dampening it^41,42^. Calves are highly motivated to suckle, especially when they are fed a restricted diet as in the current study, which is typical in the U.S. dairy industry, but is less than what they would consume ad libitum^43^. It is possible this motivation was strong enough to override a pre-existing treatment difference in risk aversion, and a different experimental design may have produced different results. For example, chicks startled near the end of a feeding bout have a greater response than those startled at its beginning, which the authors attributed to the motivation to feed initially being high at the beginning of a bout and then decreasing as the animal becomes satiated^44,45^. It would be of interest to assess how disbudding affects the startle response when calves’ motivation to feed is less dominant, or when they are asked to trade off a different motivation (e.g., access to forage or a social companion).

Certain other factors may have limited our ability to detect differences across treatments. Calves received analgesia at the time of disbudding, which may have mitigated development of a risk-averse state. In squids, anesthesia at the time of injury abolished the subsequent increase in defensive behaviour, suggesting that the acute nociceptive response is responsible for inducing this behavioural sensitization^4^. It is also likely that testing each calf alone induced anxiety^46^, which may have reduced our ability to detect treatment differences.

### Future directions

The findings from this study contradicted our hypothesis, and in the previous section we offered a post-hoc interpretation that should be tested in future experiments. However, other scientifically plausible narratives exist and deserve consideration. For example, it is possible that the greater amount of suckling following startle in disbudded calves was driven by a general increase in risk proneness, and less a heightened motivation to suckle. Although seemingly counterintuitive, risky decisions following injury may be adaptive. Life-history theory predicts that individuals should adjust their risk-taking behaviour to their expected future fitness^47^. When expectations decrease, such as following an injury, an individual should be more risk-prone as they have less to lose and more to gain. Indeed, humans and rodents suffering from chronic pain make riskier decisions than their healthy counterparts^48,49^. Thus, the greater time spent suckling in disbudded calves may reflect a life-history strategy in which they accept more risk to recoup fitness after suffering an injury.

It is also possible that unique selection pressures shape responses to injury in early life. Early injury may signal to an individual that they live in an environment with high or unpredictable risk; under such conditions, increased risk avoidance may lead to reduced fitness through lost foraging opportunities^50^. Thus, animals must be willing to take greater risks to meet energetic demands. Intriguingly, injury decreased responses to a simulated predator threat in hatchling squid, while producing the predicted increase in their adult counterparts^51^. This is consistent with reports that early predator exposure results in more risk-prone individuals than in predator-free environments^52^. A fuller understanding of the behavioural consequences of injury will require testing how decision-making varies with the nature of the perceived risk and reward, and how life-history stage influences these trade-offs.

It is difficult to draw conclusions about the welfare implications of our findings, as the motivating factors behind the disbudded calves’ decision to prioritize suckling over risk avoidance require elucidation. However, if this decision was indeed driven by a motivation to seek analgesia, this would corroborate our previous findings that calves experience ongoing pain weeks after disbudding^17,18,53^, and that current best practice for pain management (i.e., a cornual nerve block and NSAID at the time of the procedure) is insufficient. Long-term solutions, such as breeding polled (i.e., naturally hornless) animals^54,55^ or alerting husbandry systems to accommodate horned animals^56^, would avoid the need for this invasive procedure.

## Conclusion

Contrary to our hypothesis that injury increases risk aversion, we found that disbudded calves accepted more risk in order to suckle compared to uninjured controls, possibly as a strategy to mitigate pain. The motivation to suckle was more pronounced in calves with older wounds than in those who were recently disbudded, perhaps because they had longer to learn any association between suckling and pain relief. To our knowledge, this is the first study to demonstrate that disbudding influences motivational states weeks after the procedure. Previous disbudding studies generally find that overt behavioural changes disappear after a day or two, leading to potentially erroneous conclusions about the duration of its effects^57^. Our results suggest that staging behavioural trade-offs may be a promising paradigm to probe motivational changes following injury.

## Supplementary Information

Supplementary Information 1.

Supplementary Video 1.

## Data Availability

Data are available in the Dryad Digital Repository: https://doi.org/10.25338/B8XW63.
