# Supplementary material for: Injury alters motivational trade-offs in calves during the healing period
Source: Sci Rep. 2021 Mar 25;11:6888. doi: 10.1038/s41598-021-86313-z (PMC7994642; doi:10.1038/s41598-021-86313-z)
Supplement: Supplementary file 1 — Supplementary Information 1. [file 41598_2021_86313_MOESM1_ESM.docx]

# Supplementary Information

**Injury alters motivational trade-offs in calves during the healing period**

**Sarah J.J. Adcock^1,2^, Cassandra B. Tucker^1,^***

^1^Center for Animal Welfare, Department of Animal Science, University of California, Davis 95616, USA

^2^Animal Behavior Graduate Group, University of California, Davis, 95616, USA

*cbtucker@ucdavis.edu

**Supplementary Video S1.** Calves were tested individually in an outdoor arena. A 0.4 s burst of white noise was emitted as soon as the calf approached the milk bottle, and their startle response was recorded using an accelerometer attached to the right hindfoot. Calves were habituated to the arena for 15 min daily for 3 consecutive days before testing.
